# Efficacy of a continuous wound infiltration system for postoperative pain management in gynecologic patients who underwent single-port access laparoscopy for adnexal disease

**DOI:** 10.3389/fmed.2023.1199428

**Published:** 2023-07-05

**Authors:** Jun-Hyeok Kang, Kyung A Lee, Yae Rin Heo, Woo Young Kim, E Sun Paik

**Affiliations:** ^1^Department of Obstetrics and Gynecology, Uijeongbu Eulji Medical Center, Eulji University School of Medicine, Uijeongbu, Republic of Korea; ^2^Department of Obstetrics and Gynecology, Kangbuk Samsung Hospital, Sungkyunkwan University School of Medicine, Seoul, Republic of Korea

**Keywords:** single-port access laparoscopy, adnexal disease, postoperative pain, continuous wound infiltration, intravenous patient-controlled analgesia

## Abstract

**Introduction:**

Single-port access (SPA) laparoscopy requires only one incision, unlike conventional laparoscopy. However, its umbilical incision is larger than that of conventional laparoscopy and can be vulnerable to postoperative pain. This study aimed to evaluate whether simultaneous use of a continuous wound infiltration (CWI) system and intravenous patient-controlled analgesia (IV PCA) effectively decreases surgical site pain in patients who underwent SPA laparoscopy due to gynecologic adnexal disease.

**Methods:**

A total of 371 patients who underwent SPA laparoscopy and who received IV PCA or CWI was retrospectively reviewed (combined group [CWI + IV PCA, *n* = 159] vs. PCA group [IV PCA only, *n* = 212]). To evaluate postoperative pain management, the numeric rating scale (NRS) pain score after surgery, total amount of fentanyl administered via IV PCA, and additional pain killer consumption were collected.

**Results:**

The NRS scores at 12 h (1.90 ± 1.11 vs. 2.70 ± 1.08, *p* < 0.001) and 24 h (1.82 ± 0.82 vs. 2.11 ± 1.44, *p* = 0.026) after surgery were significantly lower in the combined group than in the PCA group. The total amount of PCA fentanyl was significantly smaller in the combined group than in the PCA group (*p* < 0.001). The total quantity of rescue analgesics was smaller in the combined group than in the PCA group (*p* < 0.05).

**Conclusion:**

Combined use of the CWI system and IV PCA is an effective postoperative pain management strategy in patient who underwent SPA laparoscopy for adnexal disease.

## 1. Introduction

Single-port access (SPA) laparoscopy has been widely performed in gynecologic disease due to its advantages over conventional laparoscopy (1, 2). SPA requires only one abdominal incision at the umbilicus, unlike conventional laparoscopy, which requires at least two or three incisions. However, the umbilical incision of SPA laparoscopy is larger than that of conventional laparoscopy because insertion of all laparoscopic instruments and specimen retrieval are performed through the umbilicus (3). Therefore, patients treated with SPA laparoscopy can be vulnerable to postoperative pain despite receiving the smallest number of abdominal incisions among minimally invasive surgeries (MISs).

Postoperative pain is one of the major concerns for patients before undergoing surgery (4). Inadequately managed postoperative pain can cause various complications such as impairment of physical function and lengthened hospital stay, increasing socioeconomic costs (5). Therefore, pain management is considered one of the key elements in the Enhanced Recovery after Surgery (ERAS) protocol (6, 7). Various pain control methods such as systemic and regional analgesics have been developed (8). Traditionally, a combination of systemic analgesics based on intravenous patient-controlled analgesia (IV PCA) has been preferred for MIS as a multimodal approach. However, the side effects associated with opioids are a disadvantage of IV PCA (9). The continuous wound infiltration (CWI) system, in which a local anesthetic is continuously applied into the surgical wound, has been demonstrated to have postoperative pain–reducing effects and opioid-sparing effects in various laparotomic wounds (10, 11). However, no study has evaluated the effectiveness of a CWI system to manage postoperative pain for SPA laparoscopy for gynecologic disease, which requires the largest incision among MISs.

Therefore, the purpose of this study was to evaluate whether simultaneous use of the CWI system and IV PCA effectively decreases postoperative surgical site pain compared with IV PCA alone in patients who underwent SPA laparoscopy due to gynecologic adnexal diseases.

## 2. Materials and methods

### 2.1. Patient selection

We retrospectively reviewed patients who underwent SPA laparoscopy for adnexal disease in Kangbuk Samsung Hospital and or Uijeongbu Eulji Medical Center between January 1, 2020 and August 31, 2022. This study was approved by the Institutional Review Boards (IRBs) of Kangbuk Samsung Hospital (IRB no. 2022–12-030) and Eulji University (IRB no. 2023–02-015). Patients who met the following criteria were eligible for this study: (1) those who underwent adnexal surgery including ovarian cystectomy or salpingo-oophorectomy, (2) those who underwent IV PCA or CWI for postoperative pain control, and (3) those whose pain score at 6, 12, 24, and 48 h after surgery were adequately assessed using the numeric rating scale (NRS). We excluded patients who met one or more of the following criteria: (1) those who underwent another gynecologic surgery (such as hysterectomy or myomectomy) simultaneously, (2) those who were converted to multiport laparoscopy or laparotomy during surgery, and (3) those in which the IV PCA or CWI system was not sufficiently maintained during hospitalization because of side effects. Patients were then classified into the combined group (IV PCA and CWI system) and the PCA group (IV PCA only).

#### 2.1.1. Data collection and definition

Patient baseline characteristics including age, body mass index (BMI), and history of abdominal surgery were collected. Total operative time, perioperative complications, estimated blood loss (EBL), serum hemoglobin (Hb) level difference, single-port placement time, specimen retrieval time, length of umbilical incision before and after surgery, duration of hospital stay, and pathologic diagnosis were recorded for surgical outcomes. The total operative time was defined as the time from skin incision to skin closure. EBL was calculated by the anesthesiology unit as the difference between the total amounts of suction and irrigation. Serum Hb level difference was defined as the change between preoperative Hb level and Hb level on postoperative day 1. The single-port placement time was defined as the time for which pneumoperitoneum was maintained by insufflating carbon dioxide (CO2) gas. We defined specimen retrieval time as the total time required to remove the resected tissue through the in-bag removal process. To evaluate umbilical incision enlargement due to the elastic wound retractor of the single-port system and specimen retrieval process during surgery, the longest vertical length of umbilical incision was measured before and after surgery with the wound retractor inserted (Supplementary Figure S1). The length of hospital stay was defined as the time from operation day to discharge day.

For postoperative pain outcomes, the NRS, ranging from 0 (no pain) to 10 (worst pain), was used to assess surgical site pain at 6, 12, 24, and 48 h after surgery. The total amount of fentanyl citrate administered via IV PCA during the entire hospitalization period was recorded. The timing and amount of additional pain killers such as nonsteroidal anti-inflammatory drugs (NSAIDs) or opioid drugs were also recorded. In both institutions, the routine pain management protocol after SPA laparoscopy for adnexal disease is as follows. All patients underwent IV PCA routinely at the post-anesthesia care unit as a baseline pain management method immediately after surgery. The CWI system was performed in some patients, though there were no definite clinical criteria for its use. However, before surgery, detailed counseling for the clinical benefits, risks, and cost of the CWI system was offered to patients. The patients made their own decision whether receive the CWI system in addition to baseline IV PCA, and the system was applied only to patients who consented to the procedure. For IV PCA, 900–1800 μg fentanyl citrate was mixed with 100 mL of 0.9% normal saline (detailed criteria for the dosage of fentanyl citrate according to age and body weight are described in Supplementary Table S1); it was continuously infused at a rate of 1 cc/h. When patients wanted additional infusion for pain relief, a bolus dose of 15 μg fentanyl citrate was administered with 15-min lockout time. After starting an oral diet, oral NSAIDs were administered twice a day until discharge; in the case of allergic reaction to NSAIDs, oral acetaminophen was administered three times a day. For additional pain management, NSAIDs were used first, and opioid drugs were used when necessary. Additional pain killers were administered at any time upon patient request. Patients who underwent adnexal laparoscopic surgery were usually discharged one or two days after surgery if there were no complications.

#### 2.1.2. Surgical procedure

The surgical technique of SPA laparoscopy has been described previously (12). Briefly, a small vertical transumbilical incision of 2.0- to 2.5-cm was made using the open Hasson technique. A single-port elastic wound retractor was inserted through the umbilicus (Supplementary Figures S1A,B), and the longest vertical length of umbilical incision was measured (Supplementary Figure S1C). A single multichannel cap was placed on the wound retractor (Supplementary Figure S1D). Pneumoperitoneum with CO2 at 12 mmHg was established, and laparoscopic surgery (ovarian cystectomy or salpingo-oophorectomy) was performed. The resected adnexal tissues were placed into an endo-pouch specimen retrieval bag and removed through the umbilical opening using the cold knife-in-bag tissue removal technique (Supplementary Figure S1E). At the end of the laparoscopic surgery, the length of the umbilical incision was measured again with the wound retractor inserted to evaluate possible enlargement of the incision during surgery (Supplementary Figure S1F). Then, the musculo-fascial layer was closed.

In patients receiving CWI (ON-Q PainBuster^®^, I-Flow Corporation, Halyard Health, Irvine, CA, United States), the guiding needle was inserted into the subcutaneous layer from the lower end of the vertical umbilical incision to a point 7–10 cm below it (Supplementary Figures S2A,B). A multi-hole soaker catheter was then inserted under the guidance of guiding needle and placed on the musculo-facial layer along the umbilical wound (Supplementary Figure S2C). The subcutaneous layer and skin were closed, and the catheter was connected to an elastomeric pump filled with 100 mL of 0.3% ropivacaine solution (Supplementary Figure S2D). This solution was continuously infused at a rate of 2 mL/h.

#### 2.1.3. Statistical analysis

All statistical analyzes were performed using SPSS version 25.0 (IBM SPSS Statistics for Windows, IBM Corp., Armonk, NY, United States). Normality of the data was assessed with the Shapiro–Wilk test. Data with normal distribution are presented as mean ± standard deviation (SD), while median (interquartile range, IQR) were used for data with non-normal distribution. Frequency distributions among categorical variables for the two pain control methods were compared using the chi-square test or Fisher’s exact test. Multiple linear regression analysis models were used to estimate the independent contributions of variables to postoperative pain. A value of *p* < 0.05 was considered statistically significant.

## 3. Results

**Figure 1:**
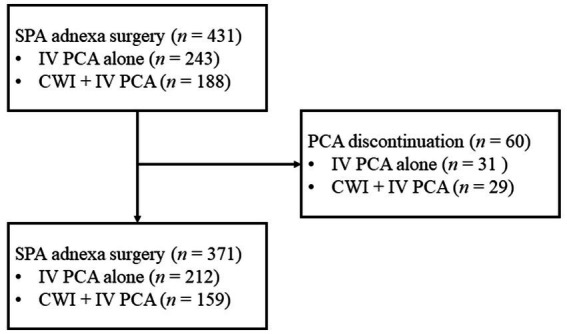
Patient selection.

During the study period, 431 patients underwent SPA laparoscopy for adnexal disease and received IV PCA or CWI for postoperative pain management. Of these, 60 patients who could not maintain IV PCA until discharge due to side effects were excluded. A total of 371 patients was enrolled in this study, comprising 212 (57.1%) patients who received only IV PCA (PCA group) and 159 (42.9%) patients who received IV PCA and CWI concurrently (combined group) (Figure 1). The patient characteristics and surgical outcomes of each group are summarized in Table 1. There was no significant difference in age, BMI, prior abdominal surgery history, length of umbilical wound before and after surgery, specimen retrieval time, operation time, and pathologic diagnosis between the two groups.

**Table 1:** Demographic characteristics and surgical outcomes.

Characteristics	Total (*n* = 371)	PCA group (*n* = 212)	Combined group (*n* = 159)	*p*-value
Age (years)	38.16 ± 13.81	37.78 ± 13.85	38.67 ± 13.78	0.541
BMI (kg/m^2^)	23.49 ± 4.12	23.80 ± 4.22	23.01 ± 3.97	0.097
ASA classification (n,%)				0.759
I	246 (66.3)	138 (65.1)	108 (67.9)	
II	117 (31.5)	69 (32.5)	48 (30.2)	
III	8 (2.2)	5 (2.4)	3 (1.9)	
Previous OP history (*n*, %)				0.241
Laparoscopy	66 (17.7)	33 (15.5)	33 (20.7)	
Laparotomy	70 (18.8)	38 (17.9)	32 (20.1)	
Operation type (n, %)				0.076
Cystectomy	254 (68.5)	153 (72.2)	101 (63.5)	
Adnexectomy	117 (31.5)	59 (27.8)	58 (36.5)	
EBL (mL)	50 (30–50)	50 (30–50)	50 (30–60)	0.462
Hb change (mg/dL)	1.95 ± 0.95	1.84 ± 0.99	2.09 ± 0.89	0.010^a^
Pre OP wound length (cm)	2.50 ± 0.06	2.50 ± 0.06	2.50 ± 0.05	0.980
Post OP wound length (cm)	2.56 ± 0.08	2.56 ± 0.09	2.57 ± 0.08	0.442
OP time (min)	56.67 ± 21.32	55.19 ± 18.86	58.65 ± 24.14	0.122
Single-port placement time (min)	34.92 ± 19.16	33.75 ± 15.98	36.45 ± 22.64	0.184
Specimen retrieval time (min)	1.59 ± 1.60	1.48 ± 0.994	1.73 ± 2.16	0.181
Hospital stay (days)	3 (2–3)	3 (2–3)	3 (2–3)	0.699
Diagnosis (n, %)				0.367
Teratoma	91 (24.5)	59 (27.8)	32 (20.1)	
Endometriosis	131 (35.3)	68 (32.1)	63 (39.6)	
Other benign conditions	135 (36.4)	78 (36.8)	57 (35.8)	
Borderline ovarian tumor	9 (2.4)	5 (2.4)	4 (2.5)	
Ovarian cancer	5 (1.3)	2 (0.9)	3 (1.9)	

PCA, Patient-Controlled Aanalgesia; BMI, Body Mass Index; ASA, American Society of Anesthesiologists physical status; OP, Operation; EBL, Expected Blood Loss; Hb, Hemoglobin.

Values are given as mean ± standard deviation or number (percentage).

a *p* < 0.05.

**Figure 2:**
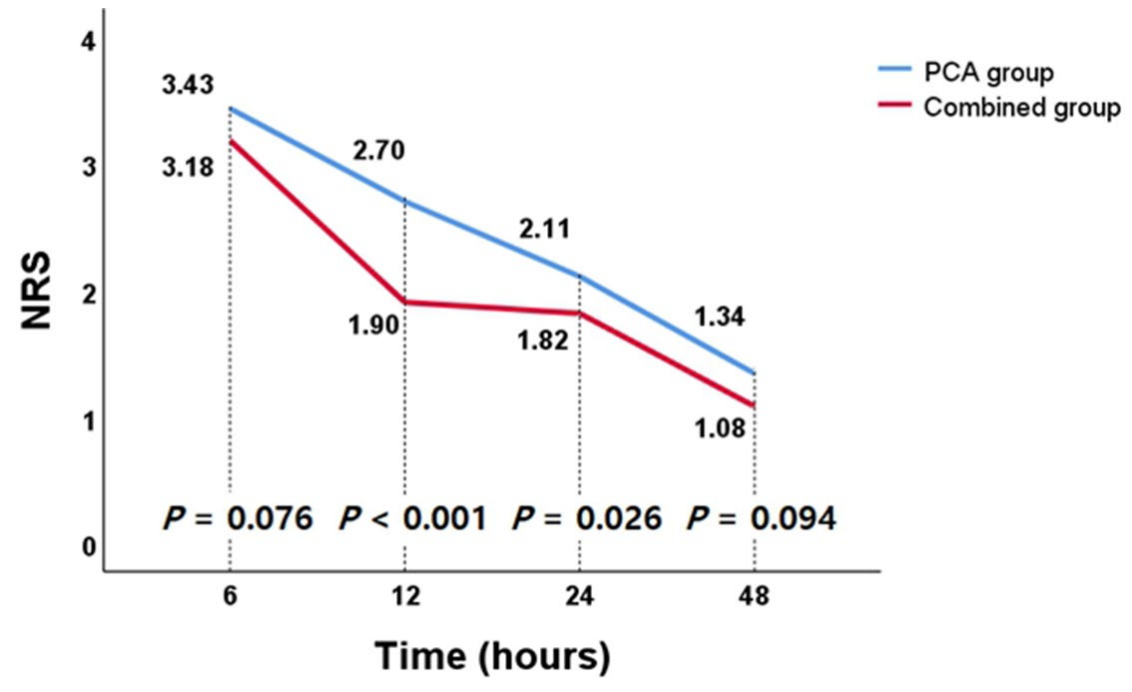
The numeric rating scale (NRS) pain scores over time after surgery in the patient groups.

The pain control outcomes according to pain management method are presented in Table 2. The NRS pain score declined gradually with time in both groups (Figure 2). The mean NRS scores assessed at 12 h (combined group vs. PCA group, 1.90 ± 1.11 vs. 2.70 ± 1.08, 95% CI [0.57; 1.03], *p* < 0.001) and 24 h (1.82 ± 0.82 vs. 2.11 ± 1.44, 95% CI [0.03; 0.55], *p* = 0.026) after surgery were significantly lower in the combined group compared with PCA group. For the remaining period, the pain intensity of the combined group tended to be lower than that of the PCA group, but it did not show statistical significance. The total amount of fentanyl citrate administered via IV PCA during the entire hospitalization period was significantly less in the combined group than in the PCA group (combined group vs. PCA group, 622.1 ± 105.3 μg vs. 703.1 ± 139.1 μg, 95% CI [56.0; 86.3], *p* < 0.001). The percentage of patients requiring rescue analgesics after surgery was lower in the combined group than in the PCA group, and in particular, there was a significant difference between 6 and 12 h after surgery (14.5% vs. 23.1%, *p* = 0.037). In addition, the CWI system significantly reduced the total amount of rescue analgesics administered from 6 to 12 h after surgery (0.23 ± 0.43 ampules vs. 0.35 ± 0.48 ampules, 95% CI [0.05; 0.22], *p* = 0.008). The use of higher potency rescue analgesics was less frequent in the combined group than the PCA group, but the difference was not significant.

**Table 2:** Pain control outcomes.

Outcomes	Total (*n* = 371)	PCA group (*n* = 212)	Combined group (*n* = 159)	*p*-value
NRS, mean ± SD				
Post OP 6 h	3.33 ± 1.35	3.43 ± 1.43	3.18 ± 1.22	0.076
Post OP 12 h	2.36 ± 1.16	2.70 ± 1.08	1.90 ± 1.11	<0.001^a^
Post OP 24 h	1.98 ± 1.21	2.11 ± 1.44	1.82 ± 0.82	0.026^a^
Post OP 48 h	1.22 ± 1.39	1.34 ± 1.51	1.08 ± 1.23	0.094
PCA fentanyl quantity (μg)	669.3 ± 123.7	703.1 ± 139.1	622.1 ± 105.3	<0.001^a^
Use of additional pain killer, *n* (%)				
Within 6 h	126 (33.9)	80 (37.7)	46 (28.9)	0.076
6–12 h	72 (19.4)	49 (23.1)	23 (14.5)	0.037^a^
12–24 h	38 (10.2)	25 (11.8)	13 (8.2)	0.256
24–48 h	19 (5.1)	12 (5.7)	7 (4.4)	0.586
Number of additional painkiller ampules used, mean ± SD				
Within 6 h	0.54 ± 0.44	0.56 ± 0.47	0.52 ± 0.42	0.425
6–12 h	0.30 ± 0.46	0.35 ± 0.48	0.23 ± 0.43	0.008^a^
12–24 h	0.12 ± 0.31	0.14 ± 0.323	0.11 ± 0.27	0.246
24–48 h	0.05 ± 0.22	0.06 ± 0.232	0.04 ± 0.588	0.558
Type of additional painkiller used, *n* (%)				
NSAIDs	157 (42.3)	90 (42.5)	67 (42.1)	0.952
Tramadol hydrochloride	48 (12.9)	32 (15.1)	16 (10.1)	0.153
Pethidine hydrochloride	27 (7.8)	20 (9.4)	7 (4.4)	0.071
Morphine sulfate	0	0	0	–

NRS, Numeric Rating Scale; PCA, Patient-Controlled Analgesia; OP, Operation; NSAIDs, Nonsteroidal Anti-Inflammatory Drugs.

Values are given as mean ± standard deviation or number (percentage).

a *p* < 0.05.

In univariate and multivariate linear regression analyzes of postoperative pain score (Table 3), postoperative wound length, operative time, single-port placement time, and pain control method were associated with pain score at postoperative 6 h. A larger postoperative umbilical wound (*β* = 2.752, *p* = 0.001), longer total operative time (β = 0.013, *p* < 0.001), and longer single-port placement time (*β* = 0.012, *p* = 0.002) were related to pain score at postoperative 6 h. However, there was no correlation between these factors and postoperative 12, 24, and 48 h pain scores. Simultaneous use of CWI and IV PCA was an independent postoperative pain-reducing factor during the entire hospitalization period in multivariate analysis.

**Table 3:** Risk factors for postoperative pain according to elapsed time after surgery.

Characteristics	Univariate				Multivariate			
	*β*	SE	*R*^2^	*p*	*β*	SE	*R*^2^	*p*-value
Post OP. 6 h								
Age	−0.09	0.005	0.008	0.088	–	–	–	
BMI	0.013	0.017	0.002	0.444	–	–	–	
Post OP wound length	2.752	0.792	0.032	0.001^a^	–	–	–	
OP time	0.013	0.003	0.039	<0.001^a^	0.013	0.003	0.051	<0.001^a^
Single-port time	0.012	0.004	0.027	0.002^a^	–	–	–	
Specimen retrieval time	0.052	0.044	0.004	0.242	–	–	–	
Pain control method^b^	−0.252	0.142	0.008	0.076	−0.306	0.142	0.051	0.032^a^
Post OP. 12 h								
Age	−0.005	0.004	0.003	0.280	–	–	–	–
BMI	0.003	0.015	0.000	0.855	–	–	–	–
Post OP wound length	0.240	0.693	0.000	0.729	–	–	–	–
OP time	0.001	0.003	0.000	0.790	–	–	–	–
Single-port time	−0.001	0.003	0.001	0.656	–	–	–	–
Specimen retrieval time	−0.025	0.038	0.001	0.506	–	–	–	–
Pain control method^b^	−0.796	0.116	0.115	<0.001^a^	−0.812	0.119	0.118	<0.001^a^
Post OP. 24 h								
Age	−0.009	0.005	0.011	0.047^a^	–	–	–	–
BMI	−0.019	0.016	0.004	0.234	-	-	-	-
Post OP wound length	−0.251	0.754	0.000	0.739	–	–	–	–
OP time	0.001	0.003	0.000	0.864	–	–	–	–
Single-port time	0.001	0.003	0.000	0.827	–	–	–	–
Specimen retrieval time	−0.005	0.041	0.000	0.904	–	–	–	–
Pain control method^b^	−0.291	0.131	0.014	0.026^a^	−0.297	0.135	0.011	0.028^a^
Post OP. 48 h								
Age	−0.013	0.006	0.015	0.026^a^	–	–	–	–
BMI	−0.011	0.018	0.001	0.554	–	–	–	–
Post OP wound length	0.342	0.895	0.000	0.703	–	–	–	–
OP time	0.005	0.004	0.005	0.183	–	–	–	–
Single-port time	0.005	0.004	0.004	0.279	–	–	–	–
Specimen retrieval time	0.031	0.047	0.001	0.520	–	–	–	–
Pain control method^b^	−0.259	0.154	0.009	0.094	−0.012	0.006	0.010	0.045^a^

BMI, Body Mass Index; OP, Operation, *R*^2^, Coefficient of Determination; *β*, Regression Coefficient; SE, Standard Error.

a *p* < 0.05.

b Pain Control Analgesia alone is the reference category.

The incidence of side effects in both patient groups is shown in Table 4. Nausea and vomiting were significantly less frequent in the combined group than the PCA group (combined group vs. PCA group, 15.9% vs. 25.0%, *p* = 0.027). Wound discharge of the umbilical incision site occurred only in the combined group maintaining CWI (21.4% vs. 0%, *p* < 0.001). After removal of CWI, wound complications such as infection and hernia were not different between the two groups.

**Table 4:** Side effects in patient groups.

Variable	Total (n = 371)	PCA group (n = 212)	Combined group (n = 159)	*p-*value
Nausea/vomiting (*n*, %)	77 (20.7)	53 (25.0)	24 (15.9)	0.027^a^
Dizziness (*n*, %)	6 (1.6)	2 (0.9)	4 (2.5%)	0.409
Hypotension (*n*, %)	4 (1.1)	1 (0.5)	3 (1.9)	0.318
Wound complication (*n*, %)				
Wound infection	10 (2.6)	7(3.3)	4 (2.5)	0.764
Wound discharge	34 (9.2)	0	34 (21.4)	<0.001^a^
Hernia	3 (0.8)	2 (0.9)	1 (0.6)	1.000

PCA, Patient-Controlled Analgesia.

Values are given as number (percentage).

a *p* < 0.05.

## 4. Discussion

This study evaluated the efficacy of the CWI system for postoperative pain management after SPA laparoscopy in patients with gynecologic adnexal disease. We found that simultaneous use of the CWI system and IV PCA (combined group) significantly reduced the immediate postoperative pain scores and the total amount of IV PCA fentanyl during the entire hospitalization. We also found that the CWI system reduces the use of additional analgesics and the incidence of adverse events such as nausea and vomiting.

Paints with gynecologic adnexal diseases may suffer from disease-specific pain before surgery and surgery-related pain after surgery. For instance, endometriosis is a representative disease that cause sever pelvic pain by creating inflammatory change, activating immune response, and increasing innervation in the affected tissue (13–15). In addition, laparoscopic surgery itself causes various types of pain such as surgical site pain, shoulder pain, and visceral pain (16, 17). MIS is considered the gold standard for benign gynecologic disease due to reduced postoperative pain, better cosmetic results, and faster recovery time compared with open surgery (18, 19). Among MISs, SPA laparoscopy is the least invasive in terms of number of abdominal scars but has the disadvantage of requiring the largest incision. Pain after abdominal surgery is generally known to be caused by the abdominal wall incision (20, 21). In laparoscopy, Ebanga et al. (22) reported that among surgical predictors of pain after laparoscopy, only fascia closure and increased operative time were related to immediate postoperative pain. In this respect, SPA laparoscopy has several potential risk factors that can aggravate surgical site pain. First, during the SPA laparoscopic procedure, at least two 5- to 10-mm laparoscopic instruments must be inserted simultaneously through a single umbilical opening. Collisions between instruments and non-ergonomic position of the surgeon can exert unintended forces on the umbilical incision. Second, unlike conventional laparoscopy, the umbilical wound is subjected to continuous tension from the elastic wound retractor of the single-port system while maintaining the pneumoperitoneum (Supplementary Figure S1B). Third, the retrieval of specimens such as huge ovarian cyst or myoma is performed through the umbilicus using an in-bag tissue removal process (Supplementary Figure S1E). During this procedure, extensive wound traction for a long time can result in enlargement of the umbilical wound. Oh et al. (23) reported that the transumbilical morcellation procedure cause umbilical incision enlargement of approximately 3 mm (from 2.5 to 2.8 cm) after surgery. In our study, wound enlargement of approximately 1 mm was observed after surgery (Table 1). This small extension can be interpreted as the fact that the adnexal tissue was relatively soft to morcellate and the specimen retrieval time was short (<2 min). The umbilical wound of SPA laparoscopy can be vulnerable to pain, for the reasons mentioned above. While previous studies have evaluated postoperative pain outcome after SPA laparoscopy, there is still no consensus on this issue (24–26). Kim et al. (26) reported that SPA surgery is more painful than conventional laparoscopy. Therefore, establishing an effective strategy to reduce pain after SPA surgery is important. Indeed, various pharmacological and technical interventions such as laparoscopic surgery under spinal anesthesia (27, 28) and bupivacaine injection to trocar sites (29) have been developed to reduce pain after laparoscopy.

The ERAS protocols to reduce hospitalization period and morbidity have become the standard for postoperative patient care after gynecologic surgery. Adequate postoperative pain management through combined use of analgesics with a different mechanism of action is a key component of the ERAS pathway. Furthermore, since the pain response to the same surgery and routine postoperative pain management for each patient, it is also important to use an appropriate rescue analgesics tailored to each patient’s characteristics (30). Recently, the 2020 American Association of Gynecologic Laparoscopists (AAGL) guidelines recommended multimodal opioid-sparing analgesia for immediate pain management after MIS (31). Opioid-based IV PCA is the preferred traditional pain control method for controlling breakthrough pain in MIS as well as open surgery. However, systemic side effects related to opioids such as nausea, vomiting, ileus, and respiratory depression are frequently reported, (32) and socio-economic costs increase due to delayed recovery and medication to alleviate these side effects. Jung et al. (33) reported that although fentanyl is an opioid with relatively few side effects, approximately 24% of patients who receive fentanyl-based IV PCA experience nausea and vomiting. In addition, according to one meta-analysis (34), opioid-based epidural PCA does not reduce recovery time compared with ERAS pain control strategy. Choi et al. (35) suggested that opioid-based IV PCA is not an essential pain control method for patients who underwent MIS for colorectal cancer. Therefore, opioid-based PCA may be inconsistent with the main purpose of the ERAS pathway, which is to reduce opioid consumption and accelerate recovery. In our study, 60 patients discontinued IV PCA due to opioid-related systemic side effects, and even in patients who maintained IV PCA until discharge, approximately 21% complained of nausea and vomiting and received additional intervention such as antiemetics. Therefore, there is need to establish an effective strategy to reduce opioid usage and postoperative pain, especially for small wounds.

The CWI system effectively controls pain by directly injecting a local anesthetic agent into the surgical site. The ERAS group also recommended that incisional infiltration has no systemic side effects when used appropriately, and it should be incorporated into other pain control methods as a component of multimodal analgesia (6). Many previous studies have demonstrated the effectiveness of the CWI system for postoperative pain control (10, 11, 36). In particular, systematic review and meta-analysis studies reported that CWI is safe and has postoperative pain reduction and opioid-sparing effects in cesarean section (37–39). However, most of these studies have focused on the large laparotomic wound and not the small laparoscopic wound. Lee et al. (40) reported that wound infiltration with short-acting local anesthetic agents effectively reduced immediate postoperative pain in the SPA laparoscopic wound. However, this one-shot administration method has a limitation in that long-term pain control is difficult due to the short half-life of the local anesthetic agent. The CWI system can be a very attractive method because it can continuously deliver a local anesthetic agent. Only a few studies have been conducted to evaluate the effectiveness of the CWI system for MIS. Oh et al. (41) evaluated the efficacy of the ropivacaine-based CWI system after laparoscopic colorectal surgery and demonstrated that it significantly reduced postoperative pain and has opioid-sparing effects. There has been no study on application of a CWI system to gynecologic SPA laparoscopy, to the best of our knowledge. In our study, simultaneous use of the CWI system and IV PCA (combined group) significantly reduced immediate postoperative pain, total amount of additional analgesia, and total quantity of opioid. In contrast, acute wound complications such as wound discharge (woozing of local anesthetic agent) have been reported only in the combined group, but there was no difference in chronic wound complications such as infection and umbilical hernia between the two groups. Furthermore, the incidence of umbilical hernia in the combined group was similar to previously reported results (0.4%) (42).

Our study demonstrated that combined use of the CWI system and IV PCA may be a safe and effective strategy to manage immediate postoperative pain in patients who underwent SPA laparoscopic adnexal surgery. Large-scale randomized trials are needed to evaluate the effectiveness of CWI in SPA laparoscopy.

To the best of our knowledge, this is the first study to evaluate the efficacy of the CWI system for gynecologic SPA laparoscopy. Another strength of this study is that it was conducted with a relatively large number of patients. However, this study has some limitations. First, this was not a prospective, randomized clinical trial. Second, among various types of pain that may occur after laparoscopic surgery such as gas-induced abdominal pain, shoulder pain, and visceral pain (16, 17), only surgical site pain was evaluated in this study. Third, there was no comparison with a CWI system-only group. Future studies should be performed to compare PCA alone, CWI alone, and a combination strategy.

## Data availability statement

The raw data supporting the conclusions of this article will be made available by the authors, without undue reservation.

## Author contributions

J-HK: conceptualization, software, visualization, and writing-original draft. J-HK and KL: data curation, investigation, and project administration. J-HK and EP: formal analysis. J-HK and WK: methodology. EP and YH: validation. KL and YH: resources. EP and WK: supervision. EP, KL, and WK: writing-review and editing. All authors contributed to the article and approved the submitted version.

## Conflict of interest

The authors declare that the research was conducted in the absence of any commercial or financial relationships that could be construed as a potential conflict of interest.

## Publisher’s note

All claims expressed in this article are solely those of the authors and do not necessarily represent those of their affiliated organizations, or those of the publisher, the editors and the reviewers. Any product that may be evaluated in this article, or claim that may be made by its manufacturer, is not guaranteed or endorsed by the publisher.
