# Supplementary material for: Efficacy of a continuous wound infiltration system for postoperative pain management in gynecologic patients who underwent single-port access laparoscopy for adnexal disease
Source: Front Med (Lausanne). 2023 Jul 5;10:1199428. doi: 10.3389/fmed.2023.1199428 (PMC10354268; doi:10.3389/fmed.2023.1199428)
Supplement: Supplementary file 1 [file Data_Sheet_1.docx]

Supplementary Material

**Efficacy of a continuous wound infiltration (CWI) system for postoperative pain management in gynecologic patients who underwent single-port access (SPA) laparoscopy for adnexal disease**

**Jun-Hyeok Kang^1†^, Kyung A Lee^2^, Yae Rin Heo^2^, Woo Young Kim^2*^, E Sun Paik^2*^**

**^*^Co-corresponding authors**

E Sun Paik

Email: esun.paik@samsung.com

Woo Young Kimz

Email: wykim.kim@samsung.com

**Supplementary Tables 1. Dosage of Fentanyl Citrate According to Age and Body Weight.**

| Body weight | **Age** | |
| --- | --- | --- |
|  | **<70 years** | **≥70 years** |
| <45 kg | 1200 µg | 900 µg |
| 45-59 kg | 1500 µg | 1200 µg |
| ≥60 kg | 1800 µg | 1500 µg |

**Supplementary Figure 1.**


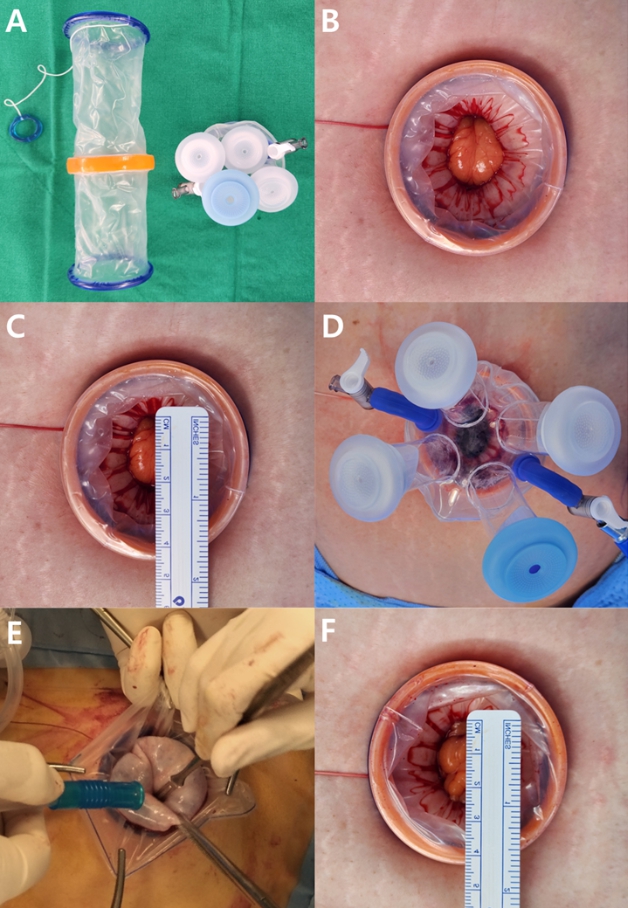


**Supplementary Figure 2.**

**
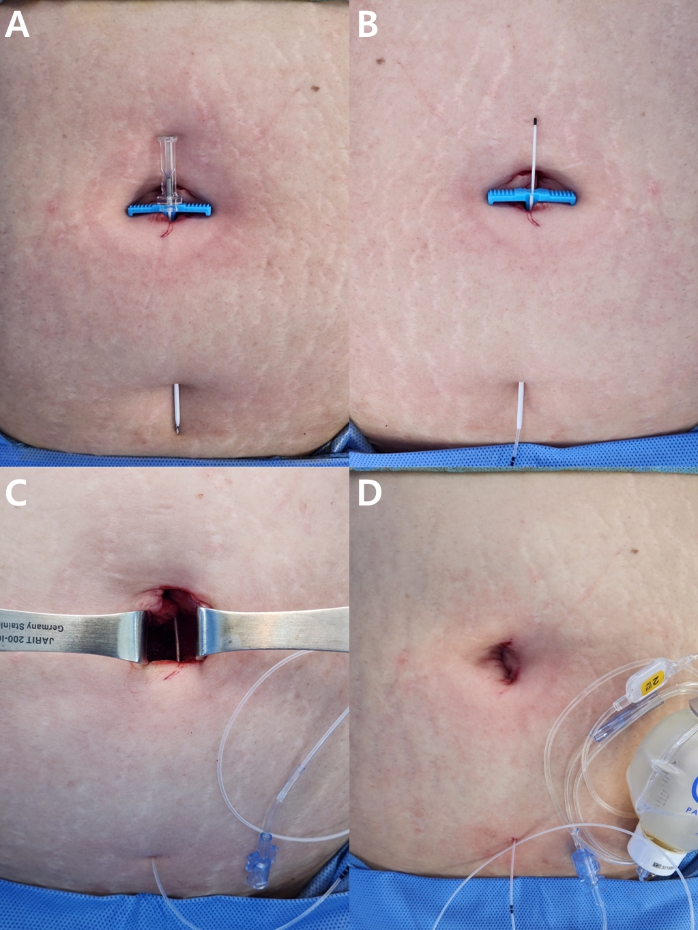
**
